# A graph-theoretic approach for classification and structure prediction of transmembrane *β*-barrel proteins

**DOI:** 10.1186/1471-2164-13-S2-S5

**Published:** 2012-04-12

**Authors:** Van Du T Tran, Philippe Chassignet, Saad Sheikh, Jean-Marc Steyaert

**Affiliations:** 1 INRIA AMIB Team, Laboratory of Computer Science (LIX), Ecole Polytechnique, 91128, Palaiseau CEDEX, France

## Abstract

**Background:**

Transmembrane *β*-barrel proteins are a special class of transmembrane proteins which play several key roles in human body and diseases. Due to experimental difficulties, the number of transmembrane *β*-barrel proteins with known structures is very small. Over the years, a number of learning-based methods have been introduced for recognition and structure prediction of transmembrane *β*-barrel proteins. Most of these methods emphasize on homology search rather than any biological or chemical basis.

**Results:**

We present a novel graph-theoretic model for classification and structure prediction of transmembrane *β*-barrel proteins. This model folds proteins based on energy minimization rather than a homology search, avoiding any assumption on availability of training dataset. The *ab initio *model presented in this paper is the first method to allow for permutations in the structure of transmembrane proteins and provides more structural information than any known algorithm. The model is also able to recognize *β*-barrels by assessing the pseudo free energy. We assess the structure prediction on 41 proteins gathered from existing databases on experimentally validated transmembrane *β*-barrel proteins. We show that our approach is quite accurate with over 90% F-score on strands and over 74% F-score on residues. The results are comparable to other algorithms suggesting that our pseudo-energy model is close to the actual physical model. We test our classification approach and show that it is able to reject *α*-helical bundles with 100% accuracy and *β*-barrel lipocalins with 97% accuracy.

**Conclusions:**

We show that it is possible to design models for classification and structure prediction for transmembrane *β*-barrel proteins which do not depend essentially on training sets but on combinatorial properties of the structures to be proved. These models are fairly accurate, robust and can be run very efficiently on PC-like computers. Such models are useful for the genome screening.

## Background

Transmembrane proteins play several key roles in the human body including inter-cell communication, transportation of nutrients, and ion transport. They also play key roles in human diseases like depression, hypertension, cancer, thus are targeted by a majority of pharmaceuticals being manufactured today. The transmembrane proteins are divided into two main types according to their conformation: *α*-helical bundles and *β*-barrels (TMB). The TMB proteins, which are much less abundant than helical bundles, are found in the outer membrane of Gram-negative bacteria, mitochondria and chloroplasts. They perform diverse functions such as porins, passive or active transporters, enzymes, defensive or structural proteins [1]. Thus, the structure of TMB proteins is very important for both biological and medical sciences.

These proteins, which span the membrane entirely, make up 20 - 30% of identified proteins in most whole genomes. However, due to difficulties in determination of their structures, solved TMB structures constitute only a meagre 2% of the RCSB Protein Data Bank (PDB) [2-5]. This is mainly due to experimental difficulties and complexity of the TMB structure [6]. Consequently, various learning-based techniques have been developed for discriminating TMB proteins from globular and transmembrane *α*-helical proteins [6-8], and for predicting TMB secondary structures [7-12]. We first discuss these methods and their potential shortcomings in detail, and then proceed with describing our approach.

Ou et al. [10] proposed a method based on radial basis function networks to predict the number of *β*-strands and membrane spanning regions in *β*-barrel outer membrane proteins. Randall et al. [9] tried to predict the TMB secondary structure with 1D recursive neural network using alignment profiles. Gromiha et al. [7,8] used the amino acid compositions of both globular and outer membrane proteins (OMPs) to discriminate OMPs and developed a feed forward neural network-based method to predict the transmembrane segments. Bagos et al. [11] produced a consensus prediction from different methods based on hidden Markov models, neural networks and support vector machines [8,13-19]. Tractability has been an issue for some of these approaches. In order to overcome this limitation, Waldispühl et al. [12] used a structural model and pairwise interstrand residue statistical potentials derived from globular proteins to predict the supersecondary structure of TMB proteins. Freeman et al. [6] have introduced a statistical approach for recognition of TMB proteins based on known physicochemical properties.

Most of these rely on the learning assumptions in the underlying models as well as the sampling of proteins in their training set. However, the number of TMB proteins known today is tiny. Thus, it is arguable whether these approaches can work well for recognizing and folding TMB proteins which are not homologous to those currently known. It is also important to note that none of these methods allow for permutations in protein structures. The TMB structures are not merely a series of *β*-strands where each is bonded to the preceding and succeeding ones in the primary sequence, but they may contain Greek key or Jelly roll motifs as well, for instance, the C-terminal domain of the PapC usher [PDB:3L48]. This level of structure may be described as a permutation on the order of the bonded strands.

In this paper, we present a novel *ab initio *model for classification and structure prediction of TMB proteins based on minimizing free energy in a graph-theoretic framework. It is able to deal with permuted TMB structures. The prediction accuracy is evaluated on known TMB proteins available in popular protein databases [20], and compared with existing software [9,10,12,21]. Our approach also performs well in structure prediction and the results are comparable to those of the existing algorithms. Ours is the first model that actually gives an insight into the physicochemical model rather than merely classifying or predicting TMB proteins. The results show that our approach is also good at discriminating TMB proteins.

## Results and discussion

### Folding

**Figure 1:**
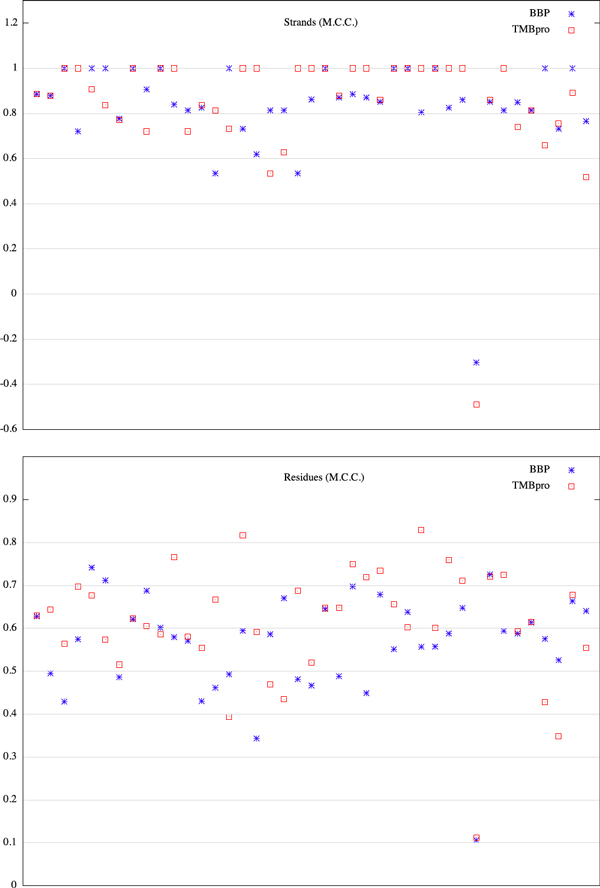
**Comparison of BBP and TMBpro on structure prediction results**.

The folding prediction results are presented in Table 1 and Figure 1. Figure 1 plots the Matthews Correlation Coefficient for our approach BBP (Beta-Barrel Predictor) and TMBpro for different proteins along the x-axis. The results of our approach are comparable to those of TMBpro but more consistent as we do not rely on training for folding. We note that, in the cases the program predicts an optimal structure with a wrong number of strands, the optimal energy is really close to the energy of the topologically right structure.

**Table 1:** Comparison of prediction accuracy on PDBTM40

	Residues					Strands			
**Method**	***Q*_2_**	**Specificity**	**Sensitivity**	**F-score**	**MCC**	**Specificity**	**Sensitivity**	**F-score**	**MCC**

*TMBpro*	81.2 ± 6.1*	79.3 * ± *7.9	84.2 ± 11.2	0.76 * ± *0.1	0.61 ± 0.14	90.1 ± 15.0	94.2 ± 12.5	0.93 ± 0.12	0.85 ± 0.26

*BBP*	79.2 * ± *5.4	78.4 * ± *6.3	80.4 * ± *9.9	0.74 * ± *0.1	0.57 ± 0.12	91.4 ± 12.0	91.4 ± 11.3	0.92 ± 0.11	0.83 ± 0.22

*Standard Deviation

The TMBETAPRED-RBF web-server predicted non-TMB for 24 over 41 proteins of PDBTM40, or 58.5%. The structures for correctly identified proteins were completely accurate. This might be because they were included in the training set.

### Evaluation of shear numbers

**Figure 2:**
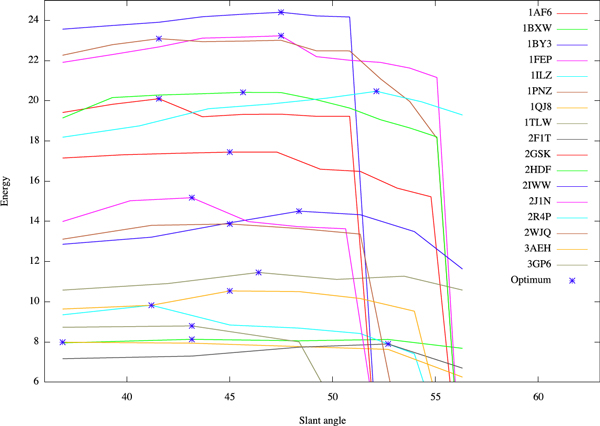
**Energy distribution of ECOLI40, **$\theta =\text{arctan}\frac{hS}{dn}$.

We study the energy distribution of 17 TMB structures (ECOLI40) in E. coli taken from PDBTM40 (including [PDB: 1AF6_A, 1BXW_A, 1BY3_A, 1FEP_A, 1ILZ_A, 1PNZ_A, 1QJ8_A, 1TLW_A, 2F1T_A, 2GSK_A, 2HDF_A, 2IWW_A, 2J1N_A, 2R4P_A, 2WJQ_A, 3AEH_A, 3GP6_A]) with regards to the slant angle, hence the shear number (see Figure 2). Most optimal structures incline with an angle of 41° - 49°, as observed in databases. This suggests that our model performs well the physicochemical properties of TMB structures. It should be also noted that there is no natural way to define the shear number *a priori*.

### Influence of the filtering threshold

We apply the filtering thresholds $\rho =\frac{1}{3},\frac{1}{2}$ and $\frac{2}{3}$on ECOLI40. These thresholds ensure that on average, considering 3-residue blocks as subunits, each segment is accepted as a *β*-strand if its propensity to be *β*-strand is at most 3, 2, 1.5 times, respectively, less than its propensity to be other structure (*α*-helices or turns/loops). The observed minor difference in accuracy with such considerably distinguished thresholds reinforces the fair independence of our approach from the training data. The results in Table 2 show the strong predicting ability of BBP from a poor known database. The lower the parameter *ρ*, the more independent to the training the predictor. This reduced the prediction performance of the model on the known structures, however, it may be useful to discover new TMB proteins.

**Table 2:** Comparison of prediction accuracy on ECOLI40 with different thresholds

	Residues					Strands			
***ρ***	***Q*_2_**	**Specificity**	**Sensitivity**	**F-score**	**MCC**	**Specificity**	**Sensitivity**	**F-score**	**MCC**

2/3	80.9 ± 4.8*	80.4 * ± *5.2	82.7 * ± *8.4	0.77 ± 0.04	0.61 ± 0.08	94.8 * ± *5.7	93.3 * ± *5.9	0.94 ± 0.05	0.88 * ± *0.1
1/2	79.7 * ± *6.0	78.5 * ± *5.1	82.4 * ± *8.6	0.76 ± 0.05	0.58 ± 0.11	96.1 * ± *4.8	95.4 * ± *5.3	0.96 ± 0.05	0.91 ± 0.09
1/3	77.7 * ± *5.6	75.6 * ± *6.5	81.1 * ± *8.6	0.74 ± 0.05	0.55 ± 0.11	91.7 * ± *9.2	94.9 * ± *6.5	0.94 ± 0.07	0.87 ± 0.07

*Standard Deviation

### Evaluation on mutated sequences

**Figure 3:**
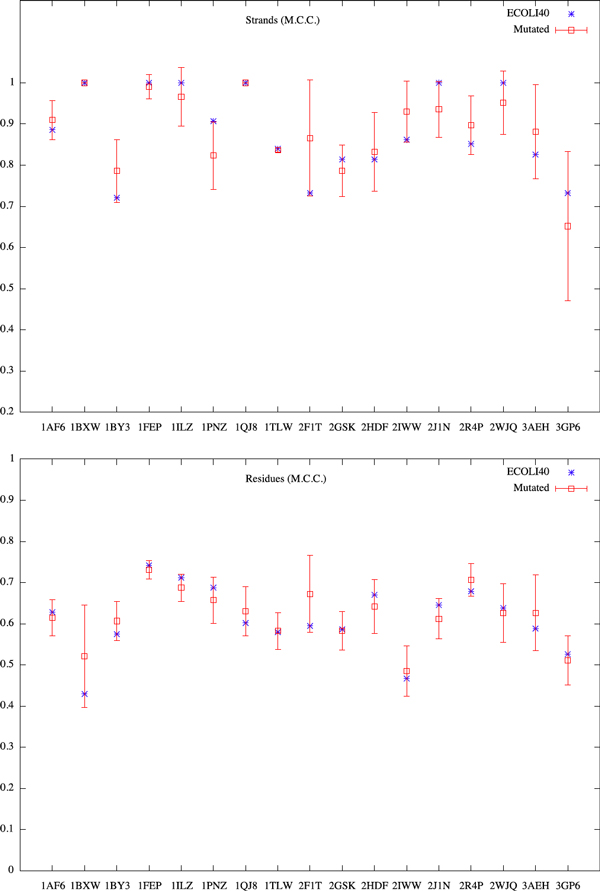
**MCC of mutated ECOLI40**.

**Figure 4:**
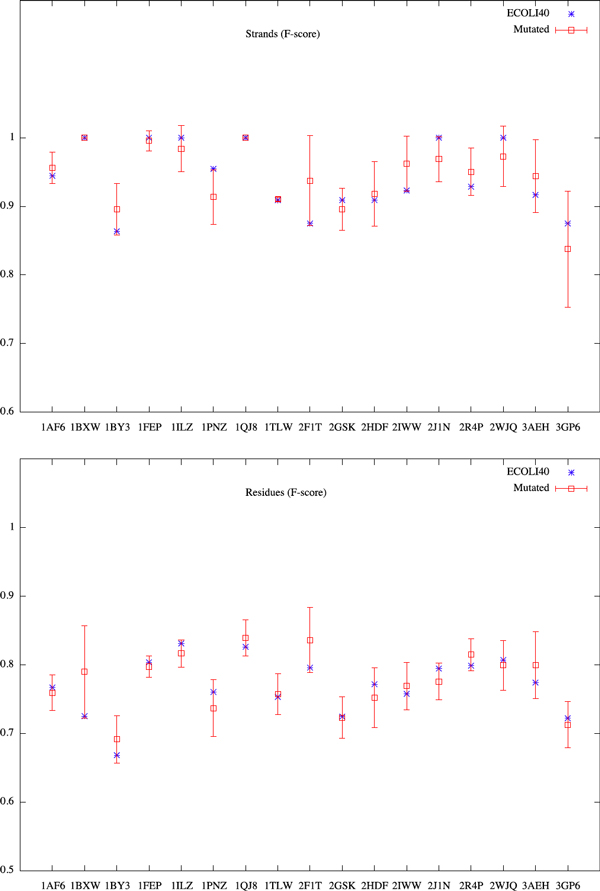
**F-score of mutated ECOLI40**.

We generate the mutated sequences from ECOLI40 by substituting the amino acids at turns or loops using the PAM250 substitution matrix [22]. Each sequence in ECOLI40 is mutated up to 5% of amino acids into 10 new sequences. Figures 3 and 4 show the Matthews Correlation Coefficient and F-score for residues and *β*-strands. We observe from these results the stability of our predictions. It also suggests that the TMB proteins are stable against these mutations at their turns and loops. The difference in structures of those mutated proteins may merely come from the shift of membrane spanning *β*-strands when their two extremities are mutated.

### Permuted structures

For [PDB:3L48], the C-terminal domain of the PapC usher in E. coli, the observed structure topology containing a Greek key motif corresponds to the permutation *σ *= (1, 4, 3, 2, 5, 6, 7) and is predicted with an accuracy (*Q*_2_) of 70.2% at *ρ *= 0.2.

**Figure 5:**
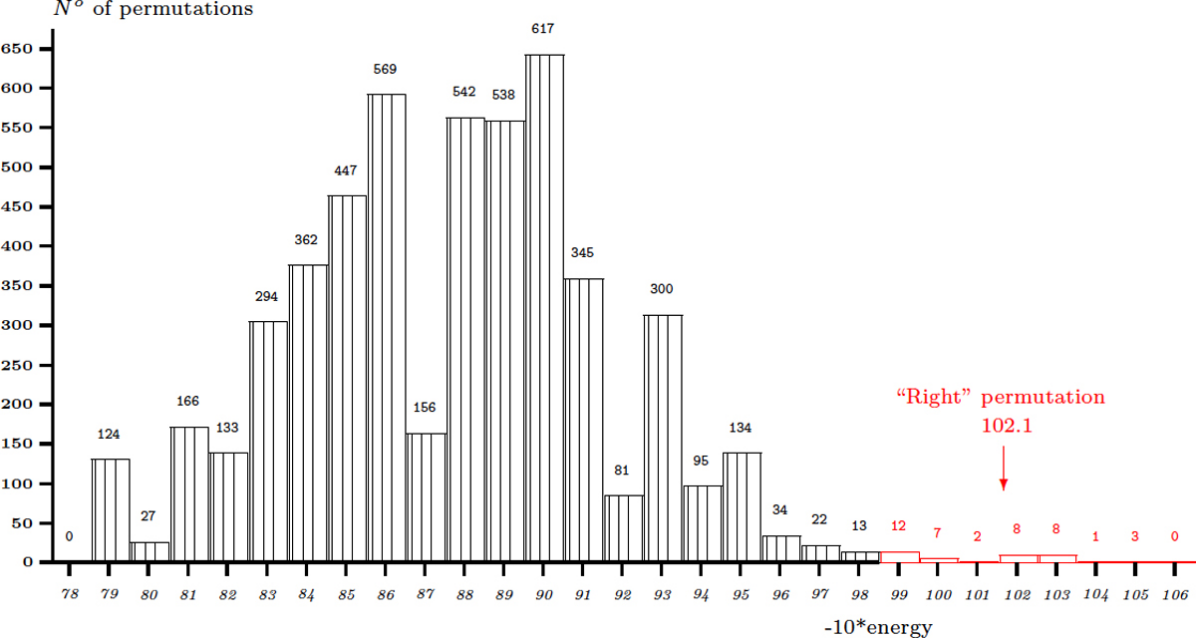
**Predicting for 7! permutations on E. Coli OmpA [PDB:**1BXW**] 8-strand barrel**.

**Figure 6:**
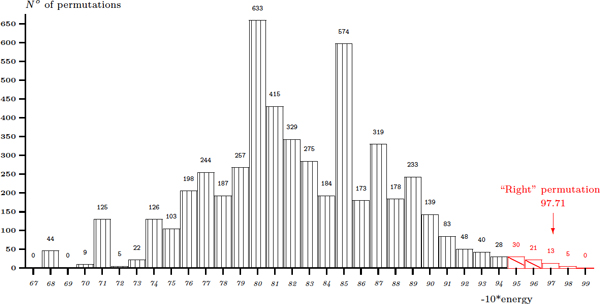
**Predicting for 7! permutations on E. Coli OmpX [PDB:**1QJ8**] 8-strand barrel.**

Following the experimental observations that were published previously on the efficiency of the *in vivo *membrane assembly of OmpA variants [23], we test our algorithm with different given permutations. OmpA [PDB:1BXW] consists of eight *β*-strands, thus without feasibility being taken into account, there are (8-1)! = 5040 circular permutations to check (see Figure 5). The pseudo-energy 10.21 of the observed permutation is found in the lowest energy zone. 41 permuted structures, or 0.81%, reach an energy of (10.21 * ± *0.3). A ratio of about 1.31% is found in the case of OmpX [PDB:1QJ8] (see Figure 6). These results are not surprising since a protein may be folded into more than one spatial conformation. In both cases, a Poisson-like distribution is found. This observation may help to discriminate most of infeasible conformations with the use of a threshold on the global energy. Hence, the method is expected to rapidly find a small set containing the right structure within a threshold of, for instance, 2% from the lowest energy and with structural feasibility conditions on permutations. This set might be much smaller be refining the biologically plausible permutations. Other proposed solutions in this set may be the candidates for *in vivo *and *in vitro *studies.

### Classification

100% of the non-redundant set of 177 *α*-helical transmembrane proteins of length from 140 to 800 residues in PDBTM are rejected, whereas 31 out of 32 non-redundant lipocalins taken from PDB are predicted as non-TMB (the dataset is available at [24]). Though lipocalins are also *β*-barrels which reverse the TMB pattern with a hydrophobic core, the environmental effects on both sides of the barrel are still different. Our pseudo-energy model yields unfavorably on such structures and discriminates considerably better than the learning-based methods like Freeman-Wimley [6], TMBpro [9], PRED-TMBB [18] and TMBETAPRED-RBF [10], but also of transFold [12].

## Conclusions

We have presented a new pseudo-energy minimization method for the classification and prediction of transmembrane protein super-secondary structure based on a variety of potential structures. Our approach takes into account many physicochemical constraints and minimizes the free energy. It also accounts for permuted structures, thus giving more complete information on the folded structure. Our method is quite accurate with more than 90% sensitivity and F-score, over 80% M.C.C. score on strands; and over 74% accuracy and F-score on residues. The results are comparable to those given by TMBpro and TMBETAPRED-RBF, which are both learning based methods. Moreover, our results are more consistent and have a significantly less variation across different TMB proteins. This is especially interesting given that our algorithm is based mainly on pseudo-energy minimizations, and the probabilistic model only plays a very small role. While the model presented here is only for TMB proteins, it can be easily extended to accommodate *α*-helical bundles. We did not use a more sophisticated statistical model for classifying *β*-barrel strands because that would risk overfitting and reliance on the training dataset. It is also interesting to note that our approach performs very well for identification of TMB proteins, rejecting all the *α*-helical bundles. The Freeman and Wimley [6] approach is more accurate on some datasets. However, it risks overfitting and does not predict the structure. Therefore, our approach provides the best overall classification results amongst the methods that try to predict structures. Our model does learn the probabilistic model from training dataset, but it is mainly to screen out obvious non-TMB strands. Therefore, there are no concerns about the size of the training data or overfitting.

Even though the results presented in this paper are comparable to other methods, the methodology presented here is novel and gives insight into the actual physicochemical constraints and energy. Moreover, our approach should be able to predict TMB proteins which are significantly different from known proteins. Finally, our approach provides more information than the current approaches by providing the permutations of the strands.

### Future work

We are working on energy models for TM *α*-helical bundles and *β*-barrels with broken strands, as well as globular *β*-barrels like lipocalins or membrane targeting proteins (C2 domain) where permuted structures are usually found. Nevertheless, similar to the other methods, we only propose single-domain protein structures.

We are also currently working on refinements in structural constraints and hydrophobicity, which may help to improve the accuracy of our predicted structure. Finally, it will be interesting to investigate more sophisticated statistical models for the initial screening, both to improve the results and understand how effective a mixed approach can be.

## Methods

We now present the methods developed for classification and structure prediction of TMB proteins (a preliminary version of this work appeared as a short paper in [25,26]). TMB proteins are hard to identify, however, it is relatively easy to identify a majority of other proteins which are not TMB. We use physicochemical properties and a simple probabilistic model based on a sliding window for filtering amino acid segments that are obviously not involved in any *β*-barrel structures as a membrane spanning *β*-strand. Proteins that are considered to be putative TMB proteins by this initial phase are then further analyzed. Next, we try to fold the given protein, treating it as a TMB protein, using the pseudo-energy minimization model. If the protein cannot be folded into *β*-barrels according to the energy minimization framework, the protein is rejected and classified as a non-TMB protein.

Before presenting the simple model that we used for filtering the transmembrane *β*-strands, we discuss some physicochemical constraints that a protein must obey to be a TMB protein. We enforce these constraints in both the filtering and folding steps of our algorithm.

### Geometric framework for *β*-barrels

For a regular *β *-barrel [27-29], the backbone geometry is entirely determined by *n*, the number of strands composing the barrel, and by *S*, the shear number, which is defined below.

Definition 1 Shear number of a *β*-barrel *In a regular β-barrel, the shear number S is unambiguously defined as the ordinal distance between an amino acid A and an amino acid B that is located on the same strand as A and linked to A through a path of hydrogen bonds. B is the projection of the "copy" of A after one turn on the first strand of the barrel*.

Structural constants are *h *(≈ 3.3Å), the jump per amino acid along a strand, and *d* (≈ 4.4Å), the mean distance between adjacent strands, given respectively by the peptide bond and hydrogen bond geometries. The other geometric characteristics, such as *θ*, the slant angle of the strands relative to the *z *barrel axis, are given from *n*, *S*, *h *and *d *[30]:

$$\text{tan}\theta =\frac{hS}{dn}$$

Angle *θ*, in association with a given membrane thickness, is involved in the energetic rules and restricts the membrane spanning *β*-strand length. Then, *n *and *S *have to be fixed as parameters.

Definition 2 Relative shear number *Given a shear number S, the *relative shears *between adjacent strands remain as n - *1 *degrees of freedom. As a convention, we consider the *relative shears *on the extracellular side of the barrel. So*, ∀*i >*1, *s_i_*, *the *relative shear *of strand i *+ 1 *with respect to strand i (strand n *+ 1 *being identified with *1*)*, *is measured on strand i as the ordinal distance between the undermost amino acid of strand i and the one that is directly bound to the undermost amino acid of strand i *+ 1.

**Figure 7:**
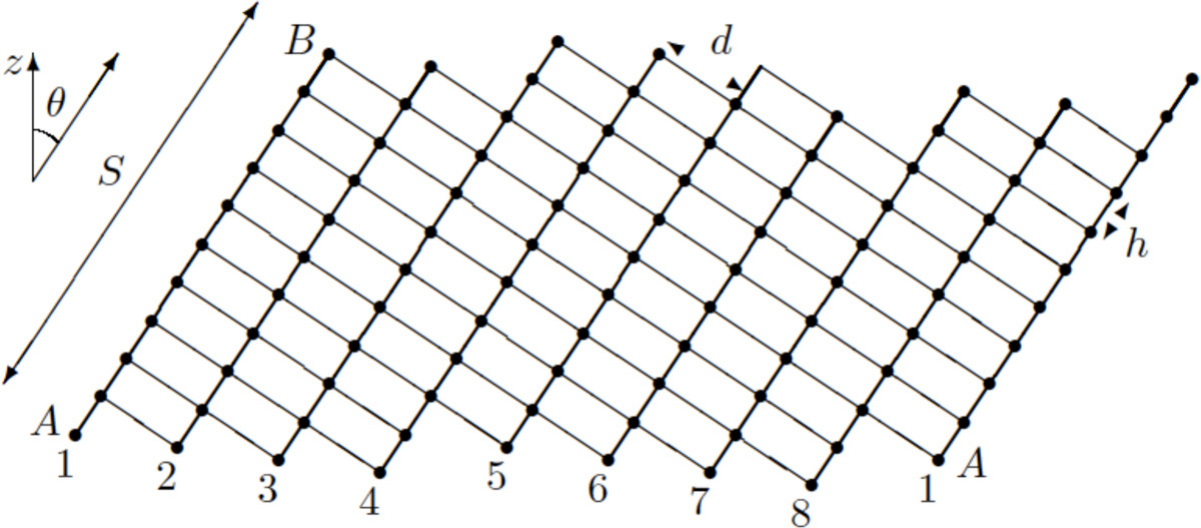
**The schematic planar view of a 8-*β *-strand barrel (strand 1 is duplicated for clarity)**. Thick lines represent the peptide bonds between consecutive amino acids along their strand. Thin lines represent the hydrogen bonds between the amino acids in adjacent strands. In this example, the *shear number *is *S *= 10, which is the ordinal distance between amino acids *A *and *B*. We note that all known *β *-barrels have a positive *shear number *[43] and are slanted "to the right", as illustrated here.

On the example of Figure 7, the sequence of *relative shears *(*s_i_*) is (1 1 1 2 1 1 1 2). The sum of consecutive *relative shears *naturally defines the *shear *between two extreme strands, thus we have the constraint for the *β*-barrel, where the two extreme strands are strand 1, for instance, and itself after a round on the barrel:

$$\underset{1\leq i\leq n}{\sum }s_{i}=S$$

We define the shear number, by extension, for the case of a *β*-sheet (i.e. an open *β*-barrel) to make our algorithms capable of dealing with the structure of *β*-sheets.

**Definition 3 Shear number of a *β*-sheet *** The shear number of a n-strand β-sheet is defined as the sum of relative shears on consecutive pairs of adjacent strands:*

$$S=\overset{}{\underset{1\leq i\leq n-1}{\sum }}s_{i}$$

*where s_i _is the relative shear of strand i *+ 1 *with regard to strand i*.

Each *β*-strand is directed with respect to the sequence order from N-terminal to C-terminal. A strand is said to be *upward *if it is oriented from the extracellular environment to the periplasmic space, i.e. the N-terminal of the strand is located on the extracellular side and its C-terminal is on the periplasmic side. Inversely, the strand is said to be *downward*. The *upward*/*downward *orientation of the strand, relatively to the barrel axis, defines another degree of freedom.

Finally, considering a *β*-strand as a ribbon where the amino acids direct their side-chains alternatively on both sides, toward the barrel interior (channel) or toward the surrounding lipid (membrane), we will distinguish two ways of facing, neglecting small swivel adjustments. A strand is said to be *odd inward *if the odd indexed amino acids face to the channel and *odd outward *if those face to the membrane. We have one more degree of freedom.

**Figure 8:**
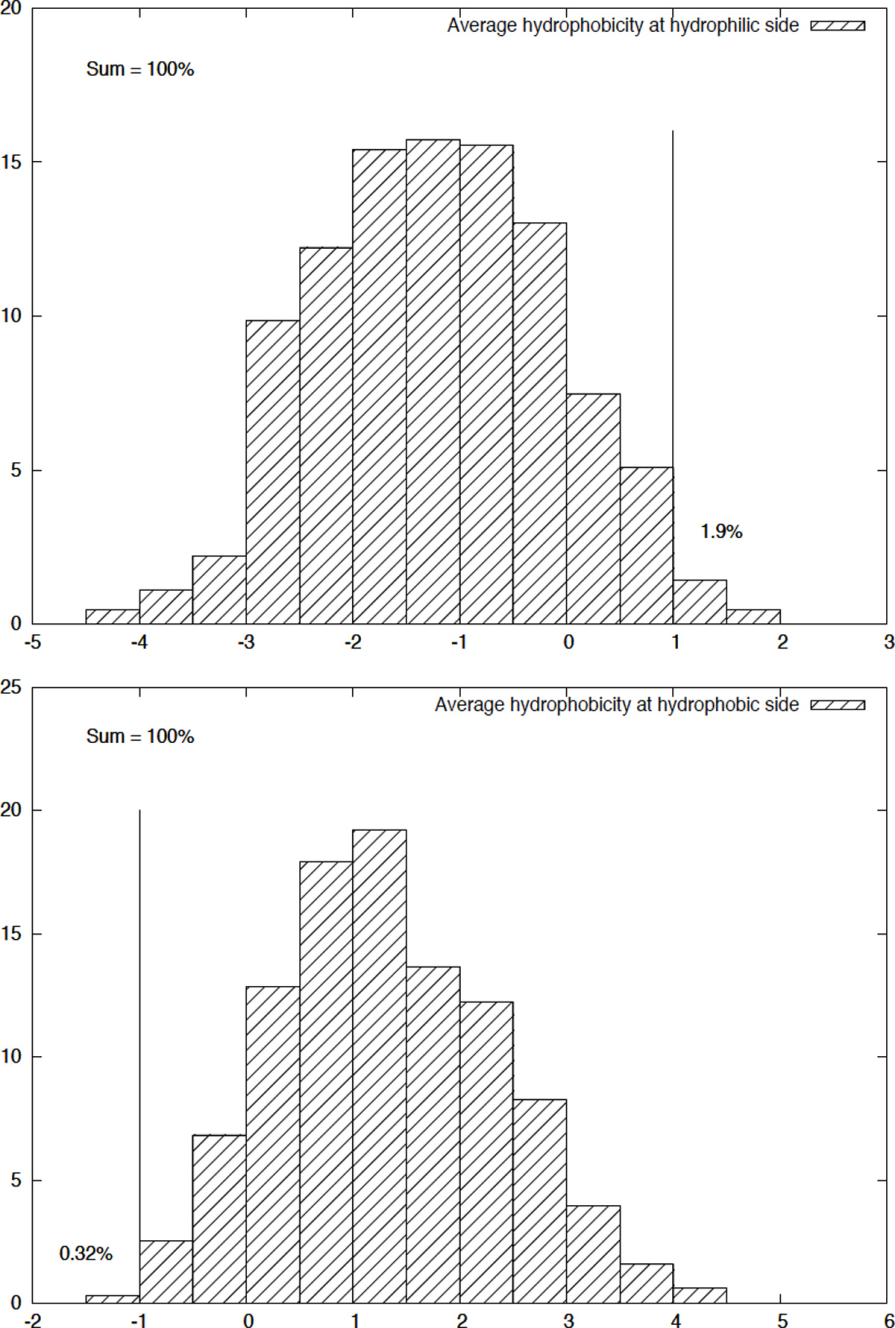
**The distribution of average hydrophobicity index of the hydrophilic and hydrophobic side of the membrane spanning *β *-strands from PDBTM40**.

**Physicochemical constraints**. On the amphipathic *β*-strand of TMB proteins, the side-chains of amino acids are directed towards the membrane and the channel alternatively. Hydrophilic and polar side-chains orient towards the aqueous interior while hydrophobic ones contact the hydrophobic bilayer [1]. We use the Kyte-Doolittle scale [31] to measure the hydrophobicity *H*(*r*) of each amino acid *r*. In this scale, a higher value represents higher hydrophobicity, and vice versa. The necessary condition for a segment *r_i _*.... *r_j _*to be a potential membrane spanning *β*-strand is that one side is hydrophobic and the other side is hydrophilic. Formally, we define

$$\begin{array}{c} H_{i,j}^{e}=\left\langle H\left(r_{2k}\right)\right\rangle ,i\leq 2k\leq j\\ H_{i,j}^{o}=\left\langle H\left(r_{2k+1}\right)\right\rangle ,i\leq 2k+1\leq j,k\in \mathbb{N} \end{array}$$

as the average hydrophobicity on the respective even and odd numbered sides. Hence, the constraints

$$\text{max}\left\{\overset{e}{\underset{i,j}{H}},H_{i,j}^{o}\right\}>\zeta^{-}\;\;\text{and}\;\;\text{min}\left\{H_{i,j}^{e},H_{i,j}^{o}\right\}<\zeta^{+}$$

are necessary for a segment of *j - i *+ 1 consecutive amino acids *r_i _*.... *r_j _*to be a potential membrane spanning *β*-strand, where *ζ ^- ^*is a lower bound for the hydrophobic side and *ζ*^+ ^is an upper bound for the hydrophilic side. We use the values *ζ ^- ^*= -1 and *ζ^+ ^*= 1, which were obtained through an statistical data analysis on known TMB structures (see Figure 8). Then, with respect to the TMB structure, the segment *r_i_*....*r_j _*is defined as *odd inward *oriented if $H_{i,j}^{o}<H_{i,j}^{e}$ and *odd outward *oriented if $H_{i,j}^{e}<H_{i,j}^{0}.$

**Classification filtering**. In order to identify substrings as potential membrane spanning *β*-strands (the vertices) or turns/loops (the edges), we introduce a simple probabilistic model that acts as a primary filter. We use a sliding window (segment) as a sequence of consecutive *l*-residue subsegments (or blocks) (*l *= 3 in our implementation). Let *r *denote the occurrence of a given block (*r *= *r*_1_*r*_2 _. . . *r_l_*) and let *τ *be the event that a block is found in a given conformation (*β*-strand or turn/loop). The information that *τ *gets from *r *is defined as:

$$I\left(\tau ;r\right)=\text{log}\frac{P\left(\tau |r\right)}{P\left(\tau \right)}=\text{log}\frac{f_{\tau ,r}/)f\cdot ,r}{f_{\tau },\cdot /)f\cdot ,\cdot },$$

where *f_τ,r _*represents the frequency observed in the training dataset for a block *r *to be found in conformation *τ *and we denote for short [32]:

$$f._{,r}=\underset{\tau }{\sum }f_{\tau ,r},\;{f_{\tau }}_{,}.=\underset{r}{\sum }f_{\tau ,r},\;f._{,}.=\underset{\tau }{\sum }\underset{r}{\sum }f_{\tau ,r}$$

Thus, *I*(*τ *; *r*) measures the influence of *r *on the occurrence of *τ*. If *I*(*τ *; *r*) = 0, there is no influence; whereas *I*(*τ *; *r*) *>*0 indicates that *r *is favorable to the occurrence of *τ *and vice versa. Formally, the preference of *r *in favor of *τ *as opposed to $\bar{\tau }$, any conformation different from *τ *[33], is:

$$I\left(\tau :\bar{\tau };\;r\right)=I\left(\tau ;\;r\right)-I\left(\bar{\tau };\;r\right)=\text{log}\frac{f_{\tau ,r}/)f_{\tau ,}.}{f_{\bar{\tau },r}/)f_{\bar{\tau },}.}$$

A simple measure is associated to each segment *r*_1 _*r*_2 _. . . *r_p _*that helps determine if it is likely a *β*-strand or a turn/loop. It is defined as the sum of informations on all the l-residue blocks:

$$Ĩ\left(\tau :\bar{\tau };r_{1}\;r_{2}\ldots r_{p}\right)=\overset{p-l+1}{\underset{i=1}{\sum }}\frac{I\left(\tau :\bar{\tau };\;r_{i}\;r_{i+1}\ldots r_{i+l-1}\right)-\text{log}\rho }{p-l+1}$$

The segment is then considered as a candidate for conformation *τ *if $Ĩ\left(\tau \;:\;\bar{\tau };r_{1}\;r_{2}\ldots r_{p}\right)>0.$

The non-redundant training set PDBTM40 of 41 TMB proteins is used to learn this probabilistic model. Due to the small size of the training set, we apply the filter with a relatively low threshold at $\rho =\frac{2}{3}$ to avoid overfitting. This ensures that on average, each block *r *is accepted in conformation *τ *if the propensity for *τ *to be in *τ *(i.e. *f_τ ,r _*/*f_τ_*, ·) is at most 1.5 times less than the propensity to be in $\bar{\tau }\left(\text{i}\text{.e}\text{.}f_{\bar{\tau },r}/f_{\bar{\tau },}\cdot \right)$. Only substrings that pass these very stringent criteria are considered to be putative strands.

Now we present a graph-theoretic energy minimization model for recognizing and folding TMB proteins.

### Definition of the graph structure

**Figure 9:**
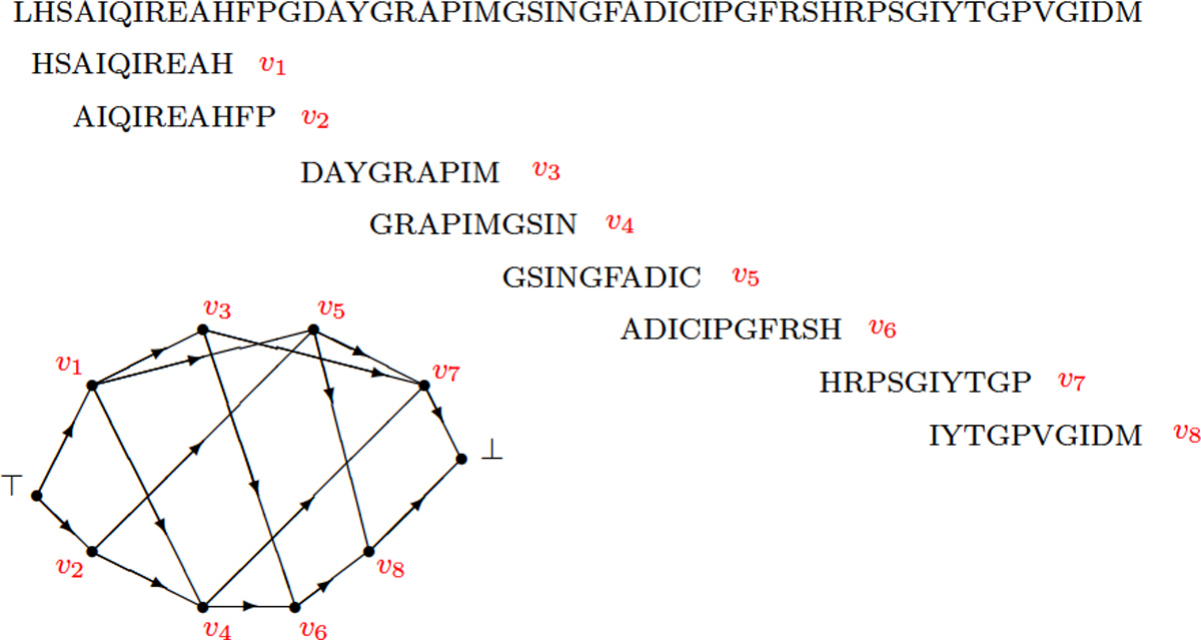
**A short example of the graph structure**.

**Dynamic programming approach**. Let S be the sequence of the *N *amino acids constituting the primary structure of a given protein. We will consider $G\left(V,E,E_{\text{intr}},E_{\text{adj}},E_{\text{loop}}\right)$, the weighted directed acyclic graph (DAG) [34] built from *S *as follows: **Vertices **Let **V **= V* ∪ {⊤, ⊥} be the set of vertices. Each vertex of V* represents a candidate secondary structure item as a *β*-strand associated with a given set of parameters. It corresponds to a contiguous part (a substring, defined by its starting and ending indices 1 ≤ *v *<* k *≤ *N*) of S that satisfies given conformational constraints (such as length, propensity to be a *β*-strand, . . .). The associated parameters provide information about the discretized spatial laying of this part relatively to the whole structure. So, combining the *upward*/*downward *and *inward*/*outward *degrees of freedom previously introduced, we consider 4 different orientations for each given candidate *β*-strand. We could also consider the different instances of *relative shear *to multiply the number of vertices, but we do not for reasons to be clarified later. A canonical order is defined on V* as the lexicographic order on tuples formed by the respective starting/ending indices in S and the associated parameters. The length constraint implies that the number of candidate substrings and thus *|***V***|*, the number of vertices, are bounded above by *kN *for a small value *k*. To simplify further definitions, a dummy vertex ⊤ will be used to represent an empty substring at the start of S and, similarly, ⊥ will represent an empty substring at the end of the sequence. To extend the order on all of the vertices, we set ⊤ <*v *< ⊥, ∀*v *∈ V* (see Figure 9).

#### Edges

Let **E **⊂ **V ***× ***V **be the set of directed edges. Intuitively, an edge corresponds to a turn or a loop that connects two consecutive *β*-strands. To be more precise, ∀*v*, *w *∈ V*, with *ν_v_*, *κ_v_*, *ν_w_*, *κ_w _*denoting their respective starting and ending indices, (*v*, *w*) is an edge, if *κ_v _*<*ν_w _- *2 and the substring of amino acids from *κ_v _*+ 1 to *ν_w _*- 1 satisfies the constraints that allow to form a turn or a loop (such as conditions on length, flexibility, propensity, . . .) also depending on the relative laying of the two substructures. We have the elementary property:

$$\forall v,\;w\in V^{*},\;\left(v,\;w\right)\in E\Rightarrow v<w$$

for the lexicographic order, and this ensures the DAG structure.

The set **E **also contains edges of the form (⊤, *v*) that define the subset of starting vertices - the leading substrings satisfying specific constraints. Similarly, **E **contains edges of the form (*v*, ⊥) that define the subset of ending vertices, with a satisfactory trailing substring. Again, the length constraints applied to the substrings associated to edges imply that *|***E***|*, the number of edges, is O(|V|) or O(N).

Figure 9 gives a small example of such a graph (to simplify, only one orientation has been considered). An edge like (*v*_1_, *v*_2_) is forbidden, since the two corresponding substrings overlap. Edges like (*v*_2_, *v*_3_) or (*v*_2_, *v*_6_) are also forbidden, since the inserted substrings are respectively too short for a turn or too long for a loop.

#### Energy attributes

The attributes that complete the definition of the graph **G **are pseudo-energy functions defined as follows:

• $\forall v\in V^{\text{*}},E_{\text{intr}}\left(v\right)$ represents the intrinsic energy of the given strand in the given orientation. This term is the sum of both the internal energy of the substructure, i.e. the interactions between its own amino acids, and the interaction energy with the environment (e.g. membrane and channel) apart from the rest of the considered protein.

Note that $E_{\text{intr}}\left(⊤\right)=E_{\text{intr}}\left(⊥\right)=0.$

• $\forall \left(v,w\right)\in V^{*}\times V^{*},E_{\text{adj}}\left(v,w,s\right)$ represents the interaction energy of the pair (*v*, *w*) when the two corresponding strands are placed side by side along the barrel, with respect to the respective orientation parameters associated to the vertices and accordingly to the *relative shear s*. The energy will take into account the number of contacts and different side-chain interactions such as the packing of hydrophobic cores and bonding abilities. Then, $\forall \left(v,w\right)\in V^{*}\times V^{*},E_{\text{adj}}\left(v,w\right)=\underset{s}{\text{min}}E_{\text{adj}}\left(v,w,s\right)$is the interaction energy of the pair (*v*, *w*) for an optimal relative shear. It is further assumed that $E_{\text{adj}}$ is defined over a superset of **E**, since we will consider the case where two adjacent strands are not consecutive along the sequence.

We also introduce the particular values:

$$E_{\text{adj}}\left(⊤,v\right)=E_{\text{adj}}\left(v,⊥\right)=0,\forall v\in V.$$

• An associated function *s*_adj _is defined such that:

• $\forall \left(v,w\right)\in V^{*}\times V^{*},E_{\text{adj}}\left(v,w,s_{\text{adj}}\left(v,w\right)\right)=E_{\text{adj}}\left(v,w\right),$which is a *relative shear *that leads to the optimal interaction energy.

An arising question is why the orientation degrees of freedom are described as a multiplicity of nodes but the *relative shear *degrees of freedom are considered when calculating the $E_{\text{adj}}$ terms. A first answer comes from the fact that wrong orientations are rather absolute and will result in pruning the sets **E **and **V **while the *shear *parameters are not so discriminative. The main reason is that we will consider "floating" parts in which adjacencies are already set, while a *relative shear *between any two parts is not yet known. In such a situation, attaching the *relative shears *to node pairs allows a significant factorization.

• ∀(*v, w*) ∈ **E**,∀*t*∈{1, 2, . . . , *n - *1} and ∀ *s - *a *relative shear*, $E_{\text{loop}}\left(v,w,t,s\right)$ is related to the intrinsic energy of the turn/loop between the strands *v *and *w *(consecutive along the sequence) when they are placed at a distance *t *along the barrel with a *relative shear s*. The distance *t *= 1 corresponds to the case where the strands are placed consecutively on the barrel, while an integer value *t >*1 will correspond to the case where *t - *1 other strands are interleaf.

To simplify, we will also use $E_{\text{loop}}\left(⊤,v\right)$ or $E_{\text{loop}}\left(v,⊥\right)$ for denoting the intrinsic energy of the outer fragment attached respectively to a starting or an ending vertex *v*. As such a fragment has a free side, the position parameters may be dropped.

Then, in the usual case of two *β*-strands that fold as a hairpin, the related energy is considered to be $E_{\text{adj}}\left(v,w\right)+E_{\text{loop}}\left(v,w,1,s_{\text{adj}}\left(v,w\right)\right)$. It is supposed a relative flexibility for turns and loops, so, when a fold is feasible, $E_{\text{loop}}$ is weak compared to $E_{\text{adj}}$ and the relative placement of the two *β*-strands is enforced to be close to *s*_adj_. Nevertheless, $E_{\text{loop}}$ will result in a strong penalty in the case of an unfeasible turn or loop, for example a loop with a majority of hydrophobic residues.

#### Protein folding problem

Given a graph $\text{G}\left(\text{V},\text{E},E_{\text{intr}},E_{\text{adj}},E_{\text{loop}}\right)$ defined as above, two integers *n, S*, and a permutation σ as 3 parameters, we look for the path P in **G **that maximizes the following objective function:

$$E=\underset{v\in P}{\sum }E_{\text{intr}}\left(v\right)+\underset{\left(v,w\right)\in P}{\sum }E_{1\text{oop}}\left(v,\;w\right)+\underset{\left(v,w\right)\in \sigma \left(P\right)}{\sum }E_{\text{adj}}\left(v,w\right)$$

such that $\underset{\left(v,w\right)\in P}{\sum }s_{\text{adj}}\left(v,\;w\right)=S.$

**Figure 10:**
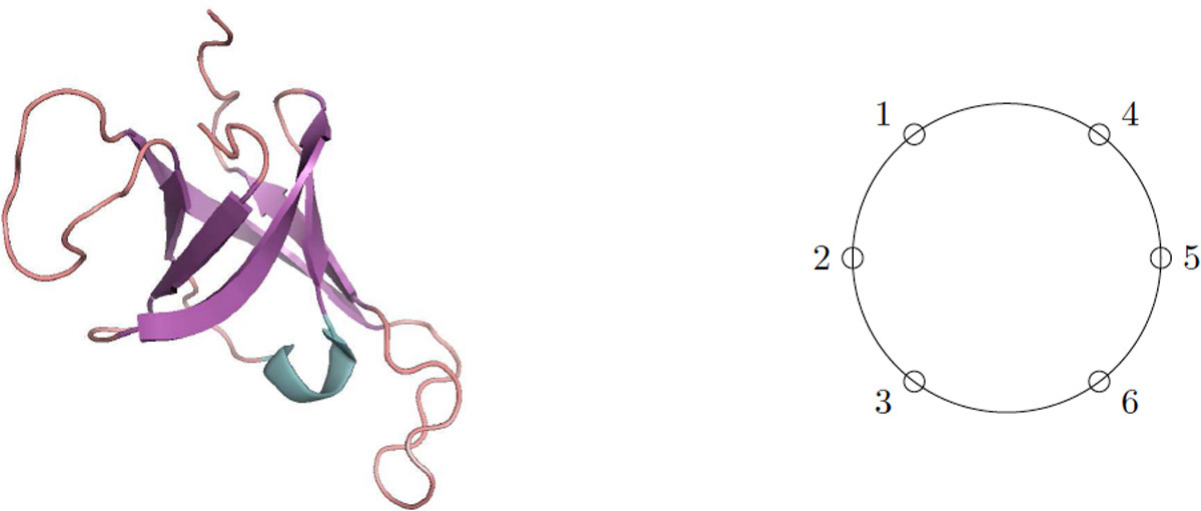
**A permuted *β*-barrel with a Greek key motif 3654, *σ *= 1 2 3 6 5 4**. Left part is a 3D synthetic view of such a protein. Right part is a schematic view from above, looking down along the axis of the barrel.

Such a path P whose vertices are arranged onto a circle is called a *circle-attached path*. The adjacent vertices in the path are not necessarily successive on the circle. This order of succession is determined by the given permutation σ (see Figure 10).

#### Solving as the longest path problem

We will first consider an open structure, as a *β*-sheet, where the adjacency of strands follows their natural order along the amino acid sequence, i.e. *σ *is an identity permutation. We involve here the constraint ∑_1<*i*≤*n *_*s_i _*= *S*. Hence, solving such a structure will result in finding a path P in **G **whose overall "energy" is given by the sum:

$$E=\Sigma_{v\in P}E_{\text{intr}}\left(v\right)+\Sigma_{\left(v,w\right)\in P}\left[E_{\text{adj}}\left(v,\;w\right)+E_{1\text{oop}}\left(v,\;w,\;1,\;s_{\text{adj}}\left(v,\;w\right)\right)\right]$$

Aiming at minimizing E, the protein folding problem will turn into finding the path from ⊤ to ⊥ that maximizes the criterion C=-E. Let $C_{v}^{h}$ be the maximum value for **C **over all the paths from ⊤ to *v*, with a shear number of *h *of the corresponding *β*-sheet, then $C_{⊤}^{0}=0$ and ∀*v *∈ **V**\{⊤}, ∀*h*, $C_{v}^{h}$ is defined as:

$$C_{v}^{h}=\underset{u\in V,\left(u,v\right)\in E}{\text{max}}\;\left[C_{u}^{h-s_{\text{adj}}\left(u,v\right)}-E_{\text{intr}}\left(v\right)-E_{\text{adj}}\left(u,\;v\right)-E_{1\text{oop}}\left(u,\;v,\;1,\;s_{\text{adj}}\left(u,\;v\right)\right)\right]$$

Since the graph is a DAG, the longest path problem is solved with a well known dynamic programming scheme [34] of complexity O(|V|) in space and O(|V|+|E|) in time, that is also O(N) for both, from the structural constraints that relate |V|, |E| and *N*. The objective is the computation of $C_{⊥}^{S}$ and the optimal structure is then reconstructed by a usual traceback post-processing. Note that, for each path, we only have to consider its last vertex, so, we have to track single index states.

For a barrel secondary structure, we have to consider a closing spatial adjacency between the last and the first strands. *σ *is still an identity permutation. The constraint on the shear number becomes ∑_1<*i*≤*n*+1 _*s_i _*= *S*. The dynamic programming scheme is almost the same as previously, except that we also have to keep track of the first vertex of any path. So, ∀*v *∈ V*, such that (⊤, *v*) ∈ **E**, let $C_{\left(v,v\right)}^{0}=-E_{\text{intr}}\left(v\right)-E_{\text{loop}}\left(⊤,\;v\right),$ then the general recurrence is: ∀*v*, *w *∈ V*, ∀*h*, such that (⊤, *v*) ∈ **E**,

$$C_{\left(v,w\right)}^{h}=\underset{u\in V,\left(u,w\right)\in E}{\text{max}}\;\left[C_{\left(v,u\right)}^{h-s_{\text{adj}}\left(u,w\right)}-E_{\text{intr}}\left(w\right)-E_{\text{adj}}\left(u,\;w\right)-E_{1\text{oop}}\left(u,\;w,\;1,\;s_{\text{adj}}\left(u,\;w\right)\right)\right]$$

and a special closing step is needed: ∀ *v *∈ V*, ∀*h*, such that (⊤, *v*) ∈ **E**,

$$C_{\left(v,\;⊥\right)}^{h}=\underset{u\in V,\left(u,⊥\right)\in E}{\text{max}}\left[C_{\left(v,u\right)}^{h-s_{\text{adj}}\left(u,v\right)}-E_{\text{adj}}\left(u,\;v\right)-E_{1\text{oop}}\left(u,\;⊥\right)\right]$$

The goal is to calculate $\underset{v,\left(⊤,v\right)\in E}{\text{max}}C_{\left(v,⊥\right)}^{S}.$Thus the scheme is of complexity $O\left(|V{|}^{2}\right)$ in space and O(|V|⋅|E|) in time, that is also $O\left(N^{2}\right)$ for both, from the structural constraints. This may produce paths of any length and the constraint of *n *strands is applied as a cut in the recurrence.

#### Generalization

In a more general case, we consider permutations to deal with the fact that the arrangements of the strands along the barrel do not necessarily follow their order along the sequence. This usually occurs with Greek key motifs or more rarely with Jelly roll motifs. Hence, the protein folding problem becomes finding the longest path P in a graph with respect to a given permutation σ, i.e. the vertices of P, seen on a circle as in Figure 10 are permuted according to σ.

Let *σ *be a circular permutation of {1, 2, . . . , *n*}. When 1, 2, . . . , *n *are numbering the positions along the barrel, values *σ *(1), *σ *(2), . . . , *σ*(*n*) will give the respective ranks of the strands in the sequence order. A position of reference along the barrel is fixed by setting *σ*(1) = 1. Figure 10 shows a first example of a structure with a Greek key motif, which is described by the permutation *σ *= (1, 2, 3, 6, 5, 4).

**Figure 11:**
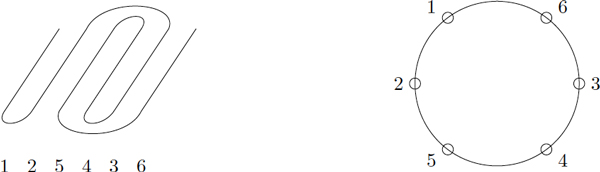
**A permuted *β*-barrel with a Greek key motif 5436, *σ *= 1 2 5 4 3 6**. Left part is a "flattened" side view, stands 6 and 1 being also adjacent. Right part is a schematic view from above, looking down along the axis of the barrel.

**Figure 12:**
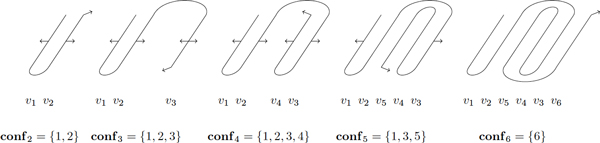
**Successive steps of the dynamic programming scheme for a Greek key motif 5436, *σ = *1 2 5 4 3 6**. Arrows show the "waiting" constraints. As all the (loop or adjacency) constraints have not been satisfied by determining the linked segments, a segment should be considered as a variable with the memory of a partial solution for each possible instance. The corresponding ranks are said "active" and are listed in the **conf** set.

Hereafter, we will illustrate the presentation of our algorithm by following the example *σ *= (1, 2, 5, 4, 3, 6), which is a bit trickier situation. This example is now said the *current example*. The corresponding structure and the dynamic programming process are illustrated in Figures 11 and 12.

The dynamic programming scheme now consists in building a barrel, by adding a next strand, taken in sequence with respect to the graph edges, but that is inserted at the position defined by the given permutation. Useful values are the ranks (in the sequence order) of the two strands between which a given one will be inserted. For instance, with the current example, the 5^th ^strand will be inserted between the 2^nd ^and the 4^th ^strands.

Let now *k *denote the level of construction (1 ≤ *k *≤ *n*), that is the number of strands already placed.

**Proposition 4 ***The k*^th ^*strand (in the sequence order) is inserted between the two strands whose ranks (in the sequence order) are ***left***_k _and ***right***_k_, defined as:*

$$left_{k}=\left\{\begin{array}{cc} \sigma \left(\sigma^{-1}\left(k\right)-1\right) & if\;\sigma^{-1}\left(k\right)>1\\ \sigma \left(n\right) & otherwise \end{array}\right),\;right_{k}=\left\{\begin{array}{cc} \sigma \left(\sigma^{-1}\left(k\right)+1\right) & if\;\sigma^{-1}\left(k\right)<n\\ 1 & otherwise \end{array}\right)$$

With the current example, we get (see Figure 11):

**left**_1 _= 6 **left**_2 _= 1 **left**_3 _= 4 **right**_1 _= 2 **right**_2 _= 5 **right**_3 _= 6

**left**_4 _= 5 **left**_5 _= 2 **left**_6 _= 3 **right**_4 _= 3 **right**_5 _= 4 **right**_6 _= 1

An important piece of information to store for the dynamic programming scheme is the set of "active" indices, i.e. ranks of the strands (in the sequence order) that are not definitively bonded on both sides, along the barrel, and also not linked along the sequence and thus have to be kept as degrees of freedom. So, in the current example (see Figure 12), we have to keep in memory as many solutions (to subproblems) as valid instances of the 2^nd ^and 4^th ^strands, until an optimal choice for these is recorded as a solution for each instance of the 5^th ^strand. At that time, any instance as the 5^th ^strand is kept as a candidate for a link with the 6^th^, by a turn or loop, while the different instances as the 3^rd ^and 1^st ^are kept for proceeding to an insertion in between.

**Definition 5 ***Two ranks i and j, which refer to the sequence order, are said "adjacent" if:*

$$|\sigma^{-1}\left(i\right)-\sigma^{-1}\left(j\right)|\;\in \;\left\{1,\;n-1\right\},$$

*where the case n *- 1 *is intended for the adjacency that will close the barrel*.

**Proposition 6 ***The set of "active" indices (in the sequence) at level k is defined by:*

$$conf_{k}=\left\{k\right\}\cup \left\{i|1\leq i<k\;\text{and}\;\left(\exists j:k<j\leq n|i,j\;are{\;}^{\prime \prime }adjecent^{\prime \prime }\right)\right\}$$

With the current example of Figures 11 and 12, we get:

**conf**_1 _= {1} **conf**_2 _= {1, 2} **conf**_3 _= {1, 2, 3}

**conf**_4 _= {1, 2, 3, 4} **conf**_5 _= {1, 3, 5} **conf**_6 _= {6}

Thus, for this example, the maximal complexity in space, $O\left(N^{4}\right)$, is reached for the set of solutions to the subproblem with 4 strands. Then looping over this set, for computing the set of solutions to the subproblem with 5 strands, will also cost $O\left(N^{4}\right)$ in time, since the choice for the 5^th ^strand is bounded by the structural constraints embedded as edges in the graph. It is a difference with most of the dynamic programming schemes where the complexity in time is expressed with an additional O(N) factor compared the complexity in space. As an other example, in the case of Figure 10, we obtain the complexity $O\left(N^{2}\right)$ in both time and space, which is similar to the case where *σ *is an identity permutation.

Now we have to decide at which minimal level *k *each term $E_{\text{adj}}$ or $E_{\text{loop}}$ is determined and can be integrated in the dynamic programming scheme. For the $E_{\text{adj}}$ terms, it is simply asserted that the previous or the next strand along the barrel is already placed when **left***_k _< k *or **right***_k _< k*, respectively.

**Proposition 7 ***For all k, we have:*

$$\begin{array}{c} left_{k}\text{ > }k\Leftrightarrow left_{k}\in conf_{k-1},\\ right_{k}<k\Leftrightarrow right_{k}\in conf_{k-1} \end{array}$$

This results from the definition of the "active" indices of **conf***_k -_*_1_. To simplify the further energy expression, we use the following notation for an "ifelse" function:

$$if_{k}\left(i,\;E\right)=\left\{\begin{array}{cc} E & \text{if}\;i<k\\ 0 & \text{otherwise} \end{array}\right)$$

For the $E_{\text{loop}}$ terms, the problem is to wait until the *relative shear *between the two ends of a turn or loop is solved by the interleaf adjacencies. So, in the given example, the energy of the loop between the 2^nd ^and 3^rd ^strands can only be evaluated when the 5^th ^strand has been laid and the optimal *relative shear *$s_{\text{adj}}^{*}\left(v_{2},v_{3}\right)=s_{\text{adj}}\left(v_{2},v_{5}\right)+s_{\text{adj}}\left(v_{5},v_{4}\right)+s_{\text{adj}}\left(v_{4},v_{3}\right)$ is known.

**Definition 8 ***Let $A_{k}$ be the relation on positive integers, defined as: *∀*i*, *j*,

$$iA_{k}j\Leftrightarrow \left[\begin{array}{c} i=j\\ or\\ i\leq k\;\text{and}\;j\leq k\;\text{and}\;i,j\;are\;{\;}^{\prime \prime }adjacent^{\prime \prime } \end{array}\right)$$

*then let *$A_{k}^{*}$ denote the equivalence relation defined by the transitive closure of $A_{k}$ and let $A_{k}=\left\{i<k|iA_{k}^{*}\left(i+1\right)\right\}.$

Thus, *i *∈ **A***_k _*means that the *i*^th ^and (*i *+ 1)^st ^strands are geometrically linked by adjacencies when the *k*^th ^substructure is laid and we can compute by composition an optimal *relative shear *$s_{\text{adj}}^{*}$.

We will now focus on the set *δ ***A***_k _*= **A***_k _*- **A***_k _*_- 1_, ∀ *k *> 1.

**Proposition 9 ***For all k, we have:*

$$\left(k-1\right)\in \delta A_{k}\Leftrightarrow left_{k}A_{k-1}^{*}\left(k-1\right)\;\;\text{or}\;\;right_{k}\;{A^{*}}_{k-1}\left(k-1\right)$$

**Proposition 10 ***For all i < k - *1,

$$i\in \delta A_{k}\Leftrightarrow \left\{\begin{array}{ccc} i\notin A_{k-1} & & \\ \text{and} & & \\ left_{k}\;A_{k-1}^{*}\;i & \text{and} & right_{k}\;A_{k-1}^{*}\left(i+1\right)\\ \text{or} & & \\ right_{k}\;A_{k-1}^{*}\;i & \text{and} & left_{k}\;A_{k-1}^{*}\left(i+1\right) \end{array}\right)$$

**Definition 11 ***Let *$T_{k}\subset {V^{*}}^{\left|conf_{k}\right|}$*denote the set of all tuples of |***conf***_k_| vertices such that there is at least one path (of k edges) starting from *⊤ *and passing through these vertices in order*.

*For any instance ***z **∈ **T***_k _of such a tuple and*, ∀*i *∈ **conf***_k_*, let **z**[*i*] *denote the i*^th ^*vertex of a corresponding path*.

This notation (not to be confused with **z***_i, _*the *i*^th ^component of tuple **z**) is not ambiguous since, from definition, the vertex **z**[*i*] is in common to any path associated to **z**. Particularly, **z**[*k*] is the last vertex of any path associated to **z**.

**Proposition 12 ***For all ***z ∈ T***_k _, the set of tuples corresponding to paths of length k - *1 *that can be extended to a path corresponding to ***z ***is defined as:*

$$pre\text{(}z\text{)}=\left\{y\in T_{k-1}|\left(y\left[k-1\right],z\left[k\right]\right)\in E\;\text{and}\;\forall i\in conf_{k}\cap conf_{k-1},y\left[i\right]=z\left[i\right]\right\}$$

Let $C_{k,z}^{h}$be the maximum value for **C **over all paths starting from ⊤ and leading in order through the vertices of a given tuple **z **∈ **T***_k _*with a shear number of *h *of the corresponding *β *-barrel. The general recurrence relation is: ∀**z **∈ **T***_k,_*

$$\begin{array}{c} C_{k,z}^{h}\;=\;\underset{y\in pre\left(z\right)}{\text{max}}\left(C_{k-1,y}^{h-s_{\text{adj}}\left(y\left[left_{k}\right],\;z\left[k\right]\right)-s_{\text{adj}}\left(z\left[k\right],y\left[right_{k}\right]\right)+s_{\text{adj}}\left(y\left[left_{k}\right],y\left[right_{k}\right]\right)}-E_{\text{intr}}\left(z\left[k\right]\right)\right)\\ -if_{k}(left_{k,}E_{\text{adj}}\text{(}y\left[left_{k}\right],z\left[k\right]-if_{k}(right_{k},E_{\text{adj}}(z\left[k\right],y\left[right_{k}\right]\\ \left(-\underset{i\in \delta A_{k}}{\sum }E_{1\text{oop}}\left(y\left[i\right],\;y\left[i+1\right],\;\sigma^{-1}\left(i+1\right)-\sigma^{-1}\left(i\right),\;s_{\text{ adj}}^{*}\left(y\left[i\right],\;y\left[i+1\right]\right)\right)\right) \end{array}$$

Note that, from proposition 7, ∀**y **∈ **T***_k _*_- 1_, if **left***_k _*<*k *then the vertex **y**[**left***_k_*] is defined (and the same is worth for **right***_k_*). We can check that each $E_{\text{adj}}$ term is finally counted exactly once in the sum, at the level corresponding to the position of its further vertex in the sequence order. The optimum is found at *k *= *n *and *h *= *S*.

**Corollary 13 ***The complexities are *$O\;\left(N^{{\text{max}}_{k}||conf_{k}||}\right)$*in space and time*.

For any permutation, we have

$$||conf_{n-k}||\;\leq \;\text{min}\;\left\{1+2k,\;n-k\right\},\;\forall k=0,\;\ldots ,\;n-1$$

Hence, max*_k _*||**conf***_k_*|| ≤ 1 + (2*n - *2) */*3. For a permutation that only differs from the identity permutation by disjoint Greek key motifs [35], i.e. $\sigma =\left(1,\;2,\;\ldots ,\;i_{1},\;G_{1},\;i_{1}+5,\ldots ,\;i_{2},G_{2},\;i_{2}+5,\ldots ,\;G_{j},\;\ldots ,\;n\right)$ where $G_{j}=i_{j}+3$, *i_j _*+ 2*, i_j _*+ 1*, i_j _*+ 4 or $G_{j}=i_{j}+1$, *i_j _*+ 4, *i_j _*+ 3, *i_j _*+ 2, it is easy to prove that max*_k _*||**conf***_k_*|| ≤ 4 by a discrete analysis on different configurations. The complexities are thus at most $O\left(N^{4}\right)$ for such a permutation.

In short, it is possible to compute the optimum in $O\left(N^{2}\right)$ running time for structures corresponding to the identity permutation and from $O\left(N^{2}\right)$ (for instance, example of Figure 10) to $O\left(N^{4}\right)$ (for instance, example of Figure 11) for structures containing disjoint Greek key motifs, where *N *is the input sequence length. These computation costs might be further improved by a tree decomposition-based algorithm that we are currently working on.

### Implementation details

The number of strands *n *and the shear number *S *determine the geometry of the barrel, particularly the membrane spanning part of the segments, and are thus involved in the computation of energy terms. If known, the algorithm can enforce these value and fold the protein accordingly. The values for *n*, which are usually even, are governed by the consideration on the length of the sequence, the thickness of membrane and the length of turns or loops and vary between 8 and 22 [1]. The values for *S*, are even and included between *n *and 2*n *[28,29]. The problem is then solved by the constrain dynamic programming with the constraints of given *n *and *S*. A small number of couples (*n, S*) have to be explored and our algorithm is fast enough for that.

Side-chain interactions between contiguous residues along a segment on the same side and interactions with the environment of channel or bilayer define the intrinsic energy of the corresponding vertex. The pairing energy of two adjacent segments in the barrel is computed by optimizing the relative positions between constituent amino acids. These energies involve hydrogen bonds in main chains, electrostatic interactions between side-chains, hydrophobic effect as well as environmental effect. More specifically, the extracellular and intracellular environments with distinct hydrophobicity indices can have significantly different hydrophobic effects. In addition, the membrane thickness gives constraints on segment size and helps identify the interactions inside or outside the membrane region. We use here by default a parameter of 3 nm for the membrane thickness, thus 8 residues thick [36,37]. The features on size, polarity [38], and flexibility [39] of turns and loops are also taken into consideration, i.e. turns and loops satisfy threshold constraints on their polarity and flexibility indices and their length. Their energies are approximated by hydrophobicity [31].

We use the Dunbrack backbone-dependent rotamer library [40] and the partial charges from GROMOS force field [41] to compute pairwise interaction energies. The hydrophobic interaction between two side-chains *u, v *is assessed by the amount of contacts between non-polar groups, calculated by taking the average on all rotamer pairs of the two side-chains *e_uv _*=*<e_uv|rotamers_>*. Each side-chain plays a role of a group of partial charges in the electrostatic interaction. The main-chain hydrogen bond is measured by the electrostatic potential energy between peptide CO and NH groups.

The probabilistic model and the constraints on hydrophobicity help discard the unlikely membrane spanning *β*-strands. A threshold on overall energy can also be involved to enhance the discrimination. We studied the per-strand energy value for a variety of TMB proteins including the training dataset and other TMB proteins. Even though this value is always higher than 0.9 for these proteins, we chose 0.85 as a threshold to avoid overfitting. Note that this does not affect the prediction results, and is only used for classification.

### Experimental setup

#### Software

We compare our folding prediction accuracy to TMBpro [9] and TMBETAPRED-RBF [10]. We compare our classification results to Freeman et al. [6], TMBETAPRED-RBF [10], PRED-TMBB [18] and transFold [12]. TMBpro and TMBETAPRED-RBF results are executed from their web-server.

#### Datasets

We used TMB proteins from the PDBTM database [20] to train and test our approaches.

• Folding: We used CD-HIT [42] to constrain the redundancy in proteins. A threshold of 40% similarity was applied to reduce the dataset, resulting in 49 sequences (PDBTM40). We retain only the monomeric barrels, i.e. the sequences that form a unique complete barrel. Thus, PDBTM40 contains 41 sequences [PDB: 1OH2_Q, 3A2R_X, 3AEH_A, 3BRZ_A, 3CSL_A, 2R4P_A, 3DWO_X, 2FGQ_X, 3EFM_A, 3EMN_X, 2ERV_A, 2IWW_A, 2F1T_A, 1FEP_A, 3FHH_A, 3FID_A, 1ILZ_A, 1BY3_A, 2GSK_A, 1BH3_A, 2HDF_A, 2J1N_A, 2IAH_A, 3JTY_A, 1BXW_A, 2VDF_A, 1PNZ_A, 3GP6_A, 1AF6_A, 3NJT_A, 2O4V_A, 2ODJ_A, 1QJ8_A, 1P4T_A, 2POR_ , 1TLW_A, 1UXF_A, 1UYN_X, 2WJQ_A, 2X4M_A, 1XKW_A]. It is important to note that both TMBPro and our method use the entire dataset to train. While this may result in overfitting for a learning-based approach, the effect on our approach should be very small.

• Classification: We used a set of 177 *α*-helical transmembrane proteins of length from 140 to 800 residues, at 40% redundancy reduction, from PDBTM and 32 non-redundant lipocalins taken from PDB.

## Competing interests

The authors declare that they have no competing interests.

## Authors' contributions

VDTT and PC designed the algorithm. JMS, SS and VDTT designed the experiments. VDTT performed the experiments and analyzed the results. VDTT, PC and SS wrote the manuscript. JMS conceived and supervised the study and revised the manuscript. All authors read and approved the paper.
